# Editorial: Long-term effects of hypoxic conditioning on sports performance, health and well-being

**DOI:** 10.3389/fphys.2022.1112754

**Published:** 2022-12-09

**Authors:** Franck Brocherie, Rafael Timon

**Affiliations:** ^1^ Laboratory Sport, Expertise and Performance (EA 7370), French Institute of Sport, Paris, France; ^2^ Faculty of Sports Sciences, University of Extremadura, Caceres, Spain

**Keywords:** altitude, resistance training, aerobic training, performance enhancement, health enhancement, inflammatory response, protein synthesis, motor unit (MU)

## Our motivation for this Research Topic

**FIGURE 1:**
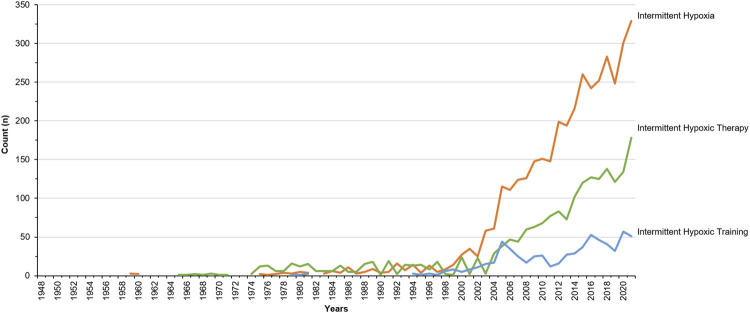
Number of publications indexed in Pubmed retrieved with the search query of key word “Intermittent Hypoxia” (in orange), “Intermittent Hypoxic Therapy” (in green) and “Intermittent Hypoxic Training” (in blue).

For decades, limiting oxygen (O_2_) availability while training has boosted physiological responses and specific adaptations, improving athletes’ performance (Millet et al., 2010). Interestingly, such a combination of hypoxia and exercise also appears relevant for health enhancement in different pathological populations. Since the 30s, the former Soviet Union and Russia have prescribed such an approach as a non-invasive, non-pharmacological treatment option (Brocherie and Millet, 2020) with few contraindications and satisfactory outcomes (Platonenko, 2017). Sharing the same acronym, intermittent hypoxic training or therapy (IHT)—also known as periodic/interval hypoxia or hypoxic (pre)conditioning, among others—has seen a growing interest in the (sports) medicine and sports sciences research community (Figure 1).

Within this Research Topic, we called for original research that may contribute to clarifying the different underlying mechanisms of hypoxic- and training-induced factors, likely to improve exercise performance, health and well-being of healthy participants (along the continuum of sport participation levels), patients with co-morbidities (*e.g.*, obesity, hypertension, diabetes) and elderly people. Five original pieces of research provide translational applications for improving exercise performance and preventing and/or treating diseases.

## Translational knowledge from performance to health (and *vice versa*)

At altitude, the reduction in O_2_ availability in the pulmonary alveoli or muscle microvasculature (Chapman et al., 2013; Calbet et al., 2016) impairs aerobic metabolism and performance (Calbet et al., 2016). This detrimental effect can be mitigated with hypoxic-based (pre)acclimatization (*i.e.*, progressive ascent, artificial hypobaric or normobaric hypoxic exposure or IHT) (Millet et al., 2010; Chapman et al., 2013). Recently, remote ischemic preconditioning (IPC)—consisting in intermittent occlusion and reperfusion of peripheral blood flow, susceptible to increase O_2_ delivery and muscle O_2_ utilization (Paradis-Deschenes et al., 2016)—potentially facilitates altitude acclimatization concomitantly with aerobic performance improvement (da Mota et al., 2019). Aiming to explore its underlying mechanisms, Zhong et al. investigated the effects of 8 consecutive days of upper arms IPC (4 × [5 min arterial occlusion 180 mm Hg/5 min reperfusion 0 mm Hg]) on aerobic performance compared to sham during acute hypobaric hypoxic exposure (462 mmHg equivalent to 4,000 m). They demonstrated that IPC alleviated the decrease in maximal O_2_ uptake, muscle and regional cerebral oxygenation caused by acute hypobaric hypoxia. Based on analytical biochemistry assay, the authors postulated that the changes observed in thymosin Tβ4, heat shock protein-70 and 90 after IPC (compared to sham) contribute to cardio-protection.

Among the recent advancement in altitude/hypoxic training, repeated-sprint training in hypoxia (RSH) has been shown to improve physical performance in various sports (Brocherie et al., 2017; Millet et al., 2019). Although most studies used normobaric hypoxia equivalent to about 3,000 m above sea level (Brocherie et al., 2017), the level of simulated altitude required to promote the greatest gains in performance remains uncertain, notably because of the large inter-individual variability in hypoxemic response to hypoxia (Woorons and Richalet, 2021). In this view, Gutknecht et al. investigated whether anaerobic performance (*i.e.,* Wingate and repeated-sprint ability) was differentially improved after RSH (*i.e.*, six sessions within 2 weeks) performed at three distinct altitude levels (*i.e.*, 1,500 m, 2,100 m and 3,200 m), and whether changes in performance were related to the pulse O_2_ saturation (SpO_2_) level achieved during RSH. Their results indicated that six sessions of RSH at altitude ≥1,500 m were effective for improving anaerobic power and capacity, but that higher altitude (at least until 3,200 m) did not provide further advantage in moderately-trained young males. Notably, no association was observed between individual SpO_2_ during RSH (ranging 78.2–94.5%) and subsequent performance changes.

Regarding endurance performance, the gains observed following hypoxic training are mainly exercise- and intensity-dependent (Millet et al., 2010; Faiss et al., 2013; McLean et al., 2014) with the high-intensity anaerobic interval method showing stronger benefits than moderate-intensity continuous exercises (McLean et al., 2014). In such cases, the hypoxic training-induced stress exacerbates the physiological and metabolic function (Mazzeo et al., 2001), with hematological changes related to immune system (Mazzeo, 2008; Born et al., 2016). Park et al., therefore, evaluated the effects of a 6-weeks IHT (3,000 m simulated altitude) on hematological parameters related to the immune system in amateur Korean female runners. Consistently with the aforementioned exercise- and intensity-dependent effect, the authors reported improvement in endurance performance, while hematological parameters related to the immune system remained in the “normal” range.

Exercising in O_2_-deprived environment opens doors as a promising therapeutic approach for targeting hypoxic-inducible factor signaling in some chronic diseases (Millet et al., 2016; Brocherie and Millet, 2020). However, depending on the patients’ condition, whether adding hypoxia with exercise or passive exposure results in health benefits remains subject to several factors, such as, for instance, hypoxic dose, exercise intensity, and/or intervention frequency. In inactive middle-aged individuals, Lizamore et al. assessed whether exercise (1 h, 3 days per week) supplemented with passive intermittent hypoxic exposure (IHE; 6 × 5 min hypoxic, 5 min normoxia; 2–3 sessions per week) over 10 weeks would improve overall cardiovascular disease risk and individual risk factors than exercise alone. Their results indicate that sequential IHE with exercise may benefit systemic markers of cardiovascular health (*i.e.*, high-density lipoprotein, systolic blood pressure and peak O_2_ uptake) but also increase myocardial load due to increased arterial wave reflection. These conflicting findings, therefore, call for caution before considering such intervention as a safe therapeutic method.

In certain cases, such as geriatric, where (poly)pharmacological treatments can lead to adverse effects due to drug interaction (Pazan and Wehling, 2021), innovative non-pharmacological strategies to prevent and treat common age-related risk diseases are most welcome. Previous IHT-related studies (*e.g.* Schega et al., 2013; Timon et al., 2021; Timon et al., 2022), have shown putative benefits for health promotion and disease prevention or treatment in elderly without elevating the mechanical stress on the musculoskeletal system. The further use of hyperoxic (fraction of inspired O_2_ = 0.30–0.40) periods during IHT (termed intermittent hypoxic-hyperoxic exposure, IHHE) (Burtscher et al., 2022) appears as a safe and well-tolerated method in geriatric patients (Behrendt et al., 2022), with the potential to boost transcription factors. Behrendt et al., therefore, investigated the acute and chronic (*i.e.*, 6 weeks of aerobic cycling training, three times a week for 20 min per day) effects of IHHE vs. sham before aerobic cycling exercise on blood lipid and lipoprotein concentrations as well as blood pressure in geriatric patients. Applying such an additional IHHE method before aerobic cycling exercise performed over 6 weeks did not influence basal lipid and lipoprotein concentrations in blood serum but seemed to be effective in reducing resting systolic blood pressure in geriatric patients of 72–94 years old.

## Closing note

The original researches included in this Research Topic promote the field-to-bench (and *vice versa*) translational implications of the chronic adaptations to non-invasive, non-pharmacological IHT-related interventions to improve exercise performance and prevent and treat several diseases. Given the inconsistent findings, possibly related to confounding factors (*e.g.*, individual variability, hypoxic mode and dose, exercise intensity), further research efforts must focus on the determination of optimal regimes and treatment protocols to improve efficiency but also safety.
